# Antiviral Used among Non-Severe COVID-19 Cases in Relation to Time till Viral Clearance: A Retrospective Cohort Study

**DOI:** 10.3390/antibiotics11040498

**Published:** 2022-04-08

**Authors:** Wael Hafez, Husam Saleh, Ziad Al Baha, Mishal Tariq, Samah Hamdan, Shougyat Ahmed

**Affiliations:** 1 NMC Royal Hospital, 16th Street, Khalifa City, Abu Dhabi P.O. BOX 35233, United Arab Emirates; husam.saleh@nmc.ae (H.S.); ziad.albaha@nmc.ae (Z.A.B.); mishal.tariq@nmc.ae (M.T.); samah.hamdan@nmc.ae (S.H.); shougyat.ahmed@nmc.ae (S.A.); 2 The Medical Research Division, Department of Internal Medicine, The National Research Center, El Buhouth Street, Cairo 12622, Egypt

**Keywords:** COVID-19, SARS-CoV-2, antiviral drugs, drug repurposing, viral clearance, symptomatic treatment

## Abstract

(1) Background: The WHO identified COVID-19 as a fast-growing epidemic worldwide. A few antivirals have shown promising effectiveness in treating COVID-19. This study aimed to assess the correlation between antiviral drugs and the time until viral clearance of SARS-CoV-2. (2) Methods: This was a retrospective cohort study that included 1731 non-severe COVID-19 patients treated in NMC Royal Hospital, UAE. (3) Results: A total of 1446 patients received symptomatic treatment only (mean age of 35.6 ± 9.0 years). The analyzed antiviral treatment protocols were azithromycin, hydroxychloroquine, lopinavir/ritonavir, and favipiravir. The produced Kaplan–Meier plots showed no significant differences in the time until viral clearance among the compared protocols, which showed overlapping confidence intervals, which were determined by performing the log-rank and adjusted pairwise log-rank tests (*p* = 0.2, log-rank = 9.3). The age and gender of patients did not significantly affect the rate of viral clearance regardless of the antiviral therapy administered, even when compared to patients who received symptomatic treatment only, with the exception of hydroxychloroquine (HCQ), azithromycin, and favipiravir, which increased the odds of a faster rate of viral clearance by 46% after adjustments. (4) Conclusions: No significant differences were observed regarding the time until viral clearance among non-severe COVID-19 patients following the prescription of different antiviral drugs.

## 1. Introduction

The Coronavirus Disease 2019 (COVID-19) outbreak caused by severe acute respiratory syndrome coronavirus-2 (SARS-CoV-2) emerged in Wuhan, China, in December 2019. On 11 March 2020, the World Health Organization (WHO) identified COVID-19 as a fast-growing epidemic worldwide. There are currently no licensed antiviral medicines for SARS-CoV-2. However, a few candidates have shown promising effectiveness as antivirals for treating COVID-19. These drugs include lopinavir/ritonavir (KALETRA^®^), hydroxychloroquine (HCQ), azithromycin (AZI), and favipiravir (AVIGAN^®^) [1].

Lopinavir antiviral is a protease inhibitor of human immunodeficiency virus 1 (HIV1) and generally used as a booster with ritonavir. Lopinavir/ritonavir can reduce protease activity and viral coronavirus replication alone or in conjunction with the interferons, both in vivo and in vitro [2,3]. Compared to the historical control of severe acute respiratory syndrome (SARS), Kaletra showed a considerably decreased risk of unfavorable clinical outcomes [4]. In vitro, lopinavir also displayed moderate antiviral activity on SARS-CoV-2 [5].

The Avigan antiviral is an inhibitor of the SARS-CoV-2 RNA polymerase that showed antiviral efficacy in vitro. This drug has previously demonstrated beneficial effects in COVID-19 patients in several trials compared to Kaletra and conventional treatments; however, one of those papers was later retracted [6,7].

Due to their immunomodulatory activities and antiviral characteristics, chloroquine and hydroxychloroquine may be an effective treatment [8,9]. The immunomodulatory effects of both drugs depend on their indirect inhibition of anti-inflammatory cytokine production, including the inhibition of interleukin (IL)-1, IL-6, interferon-γ (IFN-γ), and tumor necrosis factor. Chloroquine and hydroxychloroquine also reduce Toll-like receptor signaling, leading to both the direct and indirect inhibition of the immune system [10].

A previous observational study showed that COVID-19 patients were successfully treated with HCQ, especially when it was combined with AZI [8]. Azithromycin is an antibiotic that belongs to the macrolide family and is used to treat various bacterial diseases and has been linked to clinical outcome improvements in several viral infections, including COVID-19 [11]. AZI could play a functional role during the hyperinflammatory phase of COVID-19 via its ability to reduce cytokine production, maintain its epithelial integrity, and avoid lung fibrosis [12].

Because of the urgency of the COVID-19 epidemic, despite a lack of evidence, Kaletra and other agents have been recommended as an antiviral option for SARS-CoV-2 treatment based on the novel diagnosis and treatment guidelines released by the Chinese National Healing Commission (5th edition). Hence, using different antiviral regimens to manage COVID-19 patients may affect the time until viral clearance. For example, a previous trial showed that the time until viral clearance was similar between Kaletra therapy and conventional SARS-CoV-2 therapy. This insignificant finding may be due to the relatively late initiation of treatment after the appearance of symptoms and signs of severe complications in the participating patients [13]. Therefore, the effects of antiviral therapy should also be assessed during the early stages of the disease in non-severe COVID-19 patients.

The effectiveness of monotherapies using antivirals still requires further study and estimation. Recent studies have indicated that compared to monotherapies using antivirals, combination therapy regimens have a more significant effect on the dynamics of SARS-CoV-2 and earlier viral clearance. Hence, combined antiviral therapy could be a successful option for patients with non-severe COVID-19 [14].

This retrospective cohort study aimed to assess the correlation between the antiviral drugs used in COVID-19 treatment protocols—including hydroxychloroquine, Avigan, and Kaletra—in the United Arab Emirates (UAE) and the time until viral clearance. 

## 2. Results

### 2.1. Demographic and Clinical Characteristics of the Study Population

The study was carried out on 1794 COVID-19 patients treated in NMC Royal Hospital. There were only 1731 patients who represented non-severe cases and who were included in the analysis. We evaluated six different antiviral protocols. Moreover, about 1446 patients only received symptomatic treatment for COVID-19.

The patients were divided into seven treatment groups according to the treatments that they received, and the groups were defined as follows: a total of 81 patients (mean age = 35.9 ± 7.9 years, 5.1% and 4.6% of the total females and males, respectively) were treated with AZI only; there were 40 patients (mean age = 40.0 ± 8.9 years, 3.4% and 2.1% of the total females and males, respectively) who were treated with HCQ only; a total of 59 patients (mean age = 40.6 ± 9.5 years, 4.6% and 3.2% of the total females and males, respectively) were treated with HCQ and Avigan; there were 4 patients (mean age = 37.2 ± 6.9 years, 0.3% and 0% of the total females and males, respectively) who were treated with HCQ, Avigan, and Kaletra; a total of 60 patients (mean age = 40.2 ± 10.1 years, 3.8% and 3.4% of the total females and males, respectively) were treated with AZI and HCQ; there were 41 patients (mean age = 39.9 ± 11.4 years, 3.4% and 2.2% of the total females and males, respectively) who received AZI, HCQ, and Avigan; and 1446 patients (mean age = 35.6 ± 9.0 years, 79.7% and 84.1% of the total females and males, respectively) who received symptomatic treatment only (Table 1).

### 2.2. Time until Viral Clearance among Different Treatment Groups

**Figure 1:**
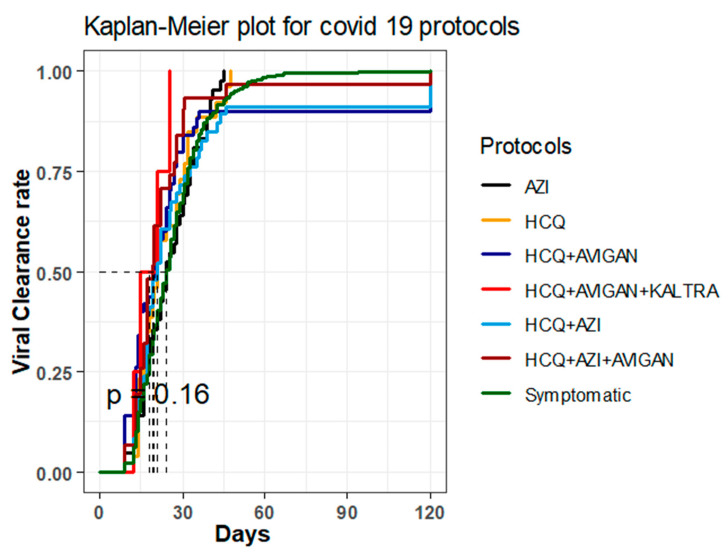
Kaplan–Meier plot for COVID-19 protocols.

The time until viral clearance was not significantly different among the different treatment groups, as shown in the Kaplan–Meier plots, which overlap between their confidence intervals and that were created by performing the log-rank test and the adjusted pairwise log-rank test (*p* = 0.2, log-rank = 9.3) (Figure 1). The median time till viral clearance was median = 24 days (95% CI, 21–31) in the AZI group, median = 21 days (95% CI, 17–31) in the HCQ only group, median = 20 days (95% CI, 15–25) in the HCQ and Avigan group, mean ± SD = 18 ± 6 days in the HCQ, Avigan, and Kaletra group, median = 21 days (95% CI, 17–26) in the HCQ and AZI group, median = 19 days (95% CI, 17–25) in the HCQ, AZI, and Avigan group, and median = 24 days (95% CI, 23–25) in the remaining patients (Figure 1).

### 2.3. The Effect of Age and Gender on Viral Clearance among Different Treatment Groups

The Cox regression model showed that the age and gender of the patients did not significantly affect the rate of viral clearance regardless of the antiviral therapy administered, even if the patients received symptomatic treatment only.

**Figure 2:**
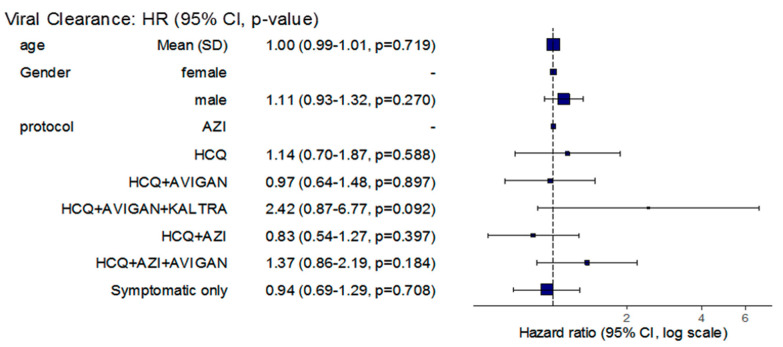
Cox regression model for age and gender of patients administered different antiviral protocols.

When the rate of viral clearance was adjusted for age and gender, the patients treated with HCQ, AZI, and Avigan were at 46% higher odds of an increased rate of viral clearance, even when compared to the patients receiving symptomatic treatment only (RR = 1.46, 95% CI: 1.01–2.10, *p* = 0.043) (Table 2) (Figure 2).

## 3. Discussion

Our findings revealed no significant differences regarding the time until viral clearance among non-severe COVID-19 patients receiving different combinations of antiviral drugs. The age and gender of the patients did not significantly affect the rate of viral clearance regardless of the antiviral therapy administered, even when compared to patients receiving symptomatic treatment only, with the exception of HCQ, AZI, and Avigan combination therapy.

Previous experience with Middle East Respiratory Syndrome (MERS) and influenza had showed that a shorter time until viral clearance contributed to improved clinical outcomes. In contrast, delayed viral clearance was related to prolonged hospitalization and poor outcomes [15]. Therefore, the association between protracted viral clearance and worse findings among patients who had been infected with SARS-CoV-2 has also been verified by several studies [16].

Persistent SARS-CoV-2 infection prolongs exposure to super-antigens that induce the systemic and chronic inflammation that has been linked to the development of chronic diseases such as diabetes and cardiovascular diseases. This inflammation could also lead to tissue damage, the activation of dendritic cells, and the induction of autoimmunity. As such, studying a patient’s viral clearance, viral load, and immune status are crucial for clinical decision-making and for determining the appropriate choice of treatment protocols [17].

Based on the results of the present investigation and previous studies, various parameters are involved in the viral clearance of SARS-CoV-2 during antiviral treatment. First, the prolonged clearance of human coronaviruses in post hematopoietic cell transplantation patients was observed to occur due to an elevated viral load [18]. Secondly, the low lymphocyte count was an independent predictor for delayed viral clearance during Kaletra and interferon-α combination therapy. These findings highlight that systemic immunomodulatory medications should be examined in patients with a high viral load and low lymphocyte count in order to strengthen the viral clearance rate [19].

A recent clinical trial showed that the median (IQR) time for SARS-CoV-2 clearance among non-survivors of COVID-19 was 18.50 (15.0:22.0) days, which was longer than the clearance time observed among survivors [18]. This observation suggests that delayed viral clearance from the respiratory tract may result in severe pneumonia. Thus, timely treatment could decrease the duration of viral clearance.

Early modeling analysis of the influenza virus showed that the antiviral and immunological response could affect viral kinetics, which involves processes where free cells are infected with the virus, and viruses are released from infected cells. Antiviral medications can prevent the virus from invading free cells and inhibit the replication of viral progeny [20]. Recent kinetic investigations have shown that the viral load of SARS-CoV-2 that is present in the respiratory system peaks at around 5–6 days after the onset of disease and remains for up to two weeks in certain patients, indicating that active intervention with antiviral agents may quicken the natural virus clearance period [21].

Similar to our findings, a systematic review and meta-analysis by Gastine et al. showed no advantage of the chloroquine/hydroxychloroquine, AZI, and Kaletra monotherapies on viral clearance rates. The authors also highlighted the possible benefits of interferons and Remdesivir in achieving faster SARS-CoV-2 clearance [22].

A recent clinical trial reported that the main endpoint, clinical improvement, had not been achieved by Kaletra. However, Kaletra has been associated with accelerated clinical recovery during modified intention-to-treat analyses (16.0 days compared to 17.0 days) (hazard ratio = 1.39; 95% CI = 1.0 to 1.91) [23].

According to a prospective study in China that included 236 COVID-19 patients with moderate symptoms, the percentage of recovery among patients receiving Avigan was more significant than it was for those receiving Arbidol treatment (71.4% vs. 55.7% *p* = 0.0199) [24]. Another observational study also showed that Avigan-receiving patients who were severely ill spent fewer days in hospital than those who were treated with Kaletra [25]. The superiority of Avigan over the standard of care to shorten the time until viral clearance was also shown by Zhao et al. [26].

A previous study found that a combination of Avigan and HCQ reduced the risk of ICU admission compared to Avigan alone. Hence, in fighting COVID-19, Avigan is regarded as the best choice [27]. Despite a considerable increase in symptom relief, Avigan was not related to significant differences in the recovery rate compared to other antivirals such as umifenavir [24].

Here, the results from the Cox’s proportional hazard model showed that patients who were treated with HCQ, AZI, and Avigan had increased odds of a faster viral clearance rate that were up to 46% higher after adjustments for age and gender.

According to a recent observational study, there is no significant evidence to prove that antimalarial drugs effectively treat COVID-19. Moreover, an international registry analysis of recently published research has retracted concerns about the safety of these medications [28]. Another clinical trial showed no difference in the SARS-CoV-2 clearance rate in patients treated who were with a combination of HCQ and Kaletra compared to the standard of care [29].

On the other hand, Chen et al. assessed the effectiveness of HCQ in 62 non-severe COVID-19 patients. The study reported faster clinical recovery and radiological improvements in the patients treated with HCQ [30]. According to a clinical trial by Gautret et al., a daily dose of 600 mg HCQ was linked to a lower viral load among COVID-19 patients. Its effect was increased when it was combined with AZI [31]. The authors verified these results with further retrospective studies of 1061 cases and have shown that both drug combinations can be linked to lower symptom recovery and fatality rates [32].

In contrast, another retrospective analysis on 1438 patients with COVID-19 revealed that HCQ, AZI, or their combination were not related to lower mortality or cardiac arrest, which are commonly detected among HCQ and AZI patients [33]. Another randomized clinical trial in Brazil showed that 397 patients with severe COVID-19 had showed no significant improvement in terms of clinical outcomes after the addition of AZI to standard care treatment (OR 1.36 [95% CI 0.94–1.97], *p* = 0.11). Hence, with the exception of being used to treat a bacterial super-infection, there is no evidence to support the usage of AZI for COVID-19 treatment [34].

According to our study, there were 1446 patients who only received symptomatic treatment. The patients had a mean age of 35.6 ± (9.0) years, with this age range including about 79.7% of female patients and 84.1% of males. The Kaplan–Meier plots showed no significant differences regarding the time until viral clearance among the compared protocols, and the plots showed overlap in terms of their confidence intervals, which was determined by performing the log-rank and adjusted pairwise log-rank tests (*p* = 0.2, log-rank = 9.3). The age and gender of the patients did not significantly affect the rate of viral clearance, regardless of the antiviral therapy administered, even when compared to the patients who received symptomatic treatment only, with the exception of those who received HCQ, AZI, and Avigan combination therapy.

Males seem to have a higher intensity and prevalence of viral infections. Females have stronger innate and adaptive immune responses than males, resulting in faster virus clearance and the development of increased immunopathology. The efficacy of antiviral medicines in reducing the viral load varies between sexes, and females are more likely than males to experience the side effects resulting from antiviral therapy. When comparing the male and female responses to viral infections and therapies, numerous variables should be considered, including hormones, genes, and gender-specific factors associated with treatment access and adherence. The baseline serum total testosterone levels were shown to significantly influence the SARS-CoV-2 clearance rate based on a recent study by Salciccia et al. Given that males and females respond differently to viruses and treatments for viral illnesses, the recommended course of action for males and females should differ [35,36].

A recent investigation by Zhou et al. examined the factors associated with the delayed clearance of SARS-CoV-2, in which prolonged viral RNA clearance was observed among patients who were younger and who had a fever, dry cough, sputum production, the presence of co-morbidities, delayed hospital admission and treatment initiation, and the development of complications. The authors also observed that treatment with Arbidol, chloroquine, and antibiotics was associated with faster viral RNA clearance; however, this association was not significant after adjusting the generalized linear model [37].

For antiviral medications to be dose-appropriate, knowledge of the potency, pharmacokinetics, and viral kinetics of acute respiratory viruses is needed to inform rational drug administration. The so-called pro-inflammatory cytokine storm generally occurs before the onset of severe disease and a poor clinical prognosis. If a patient receives treatment after having a high viral load or after the start of the cytokine storm, it is unlikely that any antiviral will be successful [38]. Besides the dose, many factors affect the conflicting results from different studies using antivirals to manage SARS-CoV-2 patients. These factors include the various antiviral protocols used, the sample size, the study design, whether the study included mixed severe and non-severe patients, confounding factors, the location of the specimen, the initial viral load, and the intervals between consecutive follow-up PCR tests for SARS-CoV-2 [39,40].

A recent mathematical modeling study showed that incomplete antiviral treatment with intermediate- to relatively high-efficacy antivirals drugs induced a prolonged time until viral clearance for SARS-CoV-2. This phenomenon could be explained by the fact that incomplete antiviral treatment decreases current infections while protecting uninfected host cells; in this case, the viral progeny that successfully escape the treatment will be capable of inducing de novo infections in the protected and uninfected cells at a slower rate, leading to a prolonged viral clearance rate [41].

The main limitation of this study was due to the limited resources available and the small sample size. Additionally, the majority of the study patients were COVID-19 patients who were mildly or moderately ill. The patients in our cohort were recruited consecutively, and most of the included patients were males and Asians, exposing our analysis to the risk of bias and chance. Additionally, we measured viral RNA using RT-PCR carried out using nasopharyngeal specimens only, which could not distinguish between viable and residual viral RNA. We did not perform a viral culture or measure the viral loads; additionally, time zero in our study was the presentation time, and all of these factors could influence our estimation of the viral clearance rate. Hence, organizations such as the WHO should take more proactive steps during this pandemic and during future epidemics and should conduct well-designed clinical trials to confirm their antiviral efficacy in the treatment of COVID-19 patients. 

## 4. Materials and Methods

### 4.1. Institutional Review Board IRB

This study was conducted according to the Declaration of Helsinki. The study was reviewed and approved by the Abu Dhabi Health COVID-19 Research Ethics Committee (Ref: DOH/CVDC/2021/1740). As a retrospective study, informed consent was not required.

### 4.2. Study Design and Study Population

This was a retrospective observational cohort study of medical records obtained from patients with non-severe COVID-19 who were treated in NMC Royal Hospital, Khalifa City, Abu Dhabi, UAE, between 8 April 2020 and 31 July 2020.

COVID-19 was diagnosed using nasopharyngeal swab specimens and Solgent’s 2019-nCoV real-time reverse transcriptase-polymerase-chain reaction (RT-PCR) kit. For RT-PCR analysis and viral identification, the Bio-Rad Cycler PCR, USA, and CFX-96 plate reader from Biorad were used per the manufacturer’s instructions. A positive SARS-CoV-2sample had a cycle threshold (CT) value greater than 40.

The testing protocol for all patients was based on the UAE’s national guidelines for the clinical management and treatment of COVID-19 [42]. All of the patients with COVID-19 symptoms were tested when they arrived at the outpatient clinic or emergency room, and then all positive cases were re-tested every 5 days, and all who showed a negative PCR test result were re-tested after 24 h. The date of viral clearance was determined by the first negative PCR test of two consecutive negatives.

The number of days between the first positive SARS-CoV-2 RT-PCR test and the first negative of two consecutive negative RT-PCR tests was used to determine the time until viral clearance. Furthermore, we classified COVID-19 patients as severe (severe/critical) or non-severe (mild/moderate) using the World Health Organization (WHO) classification criteria [43]. We assessed the correlation between the antiviral drugs used in COVID-19 protocols and the time until viral clearance. The antiviral drugs that were included were HCQ, AZI, Avigan, and Kaletra.

### 4.3. Data Management and Statistical Analysis

All data underwent statistical analysis with R Software version 3.5.2 (20 December 2018)—“Eggshell Igloo” after the protocol, the purpose of the study, and data collection and verification were discussed.

For quantitative data, mean ± standard deviation (±SD) was used for normally distributed variables. The median and interquartile range (IQR) were used when the normal distribution was violated, and the distribution was checked using the Shapiro–Wilk Test. Frequency (n) and percentage (%) were applied for the qualitative categorical variables.

Kaplan–Meier plots were created for univariate analysis to assess the differences in the time until the viral clearance of different treatment groups. For multivariate analysis, a Cox regression model was used to investigate the effect of baseline significant variables between patients who were administered different treatments. The confidence interval was 95%, and the *p*-value *p* < 0.05 was considered significant.

## 5. Conclusions

In summary, no significant differences were observed between the time until viral clearance among non-severe COVID-19 patients following different antiviral regimens. The age and gender of the patients did not significantly affect the rate of viral clearance, regardless of the antiviral therapy administered, even if the patients were only treated symptomatically, with the exception of HCQ, AZI, and Avigan, which increased the odds of a faster viral clearance rate by 46% after adjustments for age and gender. Finally, further larger, controlled, and comprehensive research investigating this combination of antivirals is needed to confirm their efficacy in the treatment of COVID-19 patients.

## Figures and Tables

**Table 1:** Baseline demographic characteristics regarding the used protocols.

		AZI ^a^ (*n* = 81)	HCQ ^b^ (*n* = 40)	HCQ + AVIGAN ^c^ (*n* = 59)	HCQ + AVIGAN + KALETRA ^d^ (*n* = 4)	HCQ + AZI (*n* = 60)	HCQ + AZI + AVIGAN (*n* = 41)	Symptomatic Only (*n* = 1446)	*p* Value
Age	Mean ± SD	35.9 ± 7.9	40.0 ± 8.9	40.6 ± 9.4	37.2 ± 6.9	40.0 ± 10.0	39.9 ± 11.4	35.6 ± 9.0	<0.001 *
Gender	female	12 (5.0%)	8 (3.4%)	11 (4.6%)	0 (0.0%)	10 (4.2%)	8 (3.4%)	189 (79.4%)	0.47
	male	69 (4.6%)	32 (2.1%)	49 (3.3%)	4 (0.3%)	51 (3.4%)	33 (2.2%)	1257 (84.1%)	

* The *p* values were calculated using the ANOVA analysis of variance for the comparative analysis between the seven treatment groups regarding the mean age of the patients, and Fisher’s exact test was used for the comparative analysis of gender. ^a^ Azithromycin (AZI), ^b^ Hydroxychloroquine (HCQ), ^c^ Favipiravir (AVIGAN®), ^d^ Lopinavir/Ritonavir (KALETRA®).

**Table 2:** Cox regression model to assess the effects of the age and gender of patients administered different antiviral protocols.

		HR (95% CI)	HR (95% CI)
Age (years)	-	1.00 (0.99–1.01, *p* = 0.931)	1.00 (0.99–1.01, *p* = 0.719)
Gender	Female	-	-
	Male	1.09 (0.91–1.30, *p* = 0.337)	1.11 (0.93–1.32, *p* = 0.270)
Protocol	AZI ^a^	-	-
	HCQ ^b^	1.14 (0.70–1.86, *p* = 0.598)	1.14 (0.70–1.87, *p* = 0.588)
	HCQ + AVIGAN ^c^	0.96 (0.63–1.46, *p* = 0.855)	0.97 (0.64–1.48, *p* = 0.897)
	HCQ + AVIGAN + KALETRA ^d^	2.48 (0.89–6.92, *p* = 0.083)	2.42 (0.87–6.77, *p* = 0.092)
	HCQ + AZI	0.84 (0.55–1.28, *p* = 0.413)	0.83 (0.54–1.27, *p* = 0.397)
	HCQ + AZI + AVIGAN	1.36 (0.85–2.16, *p* = 0.200)	1.37 (0.86–2.19, *p* = 0.184)
	Symptomatic only	0.95 (0.70–1.30, *p* = 0.754)	0.94 (0.69–1.29, *p* = 0.708)

^a^ Azithromycin (AZI), ^b^ Hydroxychloroquine (HCQ), ^c^ Favipiravir (AVIGAN®), ^d^ Lopinavir/Ritonavir (KALETRA^®^).

## Data Availability

Data are available upon request from the first and corresponding author.
